# Efficient merging of data from multiple samples for determination of anomalous substructure

**DOI:** 10.1107/S2059798315021920

**Published:** 2016-03-01

**Authors:** David L. Akey, Thomas C. Terwilliger, Janet L. Smith

**Affiliations:** a Life Sciences Institute, University of Michigan, 210 Washtenaw Avenue, Ann Arbor, MI 48109-2216, USA; b Bioscience Division, Los Alamos National Laboratory, Mail Stop M888, Los Alamos, NM 87545, USA

**Keywords:** sulfur phasing, local scaling, anomalous scattering, data merging

## Abstract

Merging of data from multiple crystals has proven to be useful for determination of the anomalously scattering atomic substructure for crystals with weak anomalous scatterers (*e.g.* S and P) and/or poor diffraction. Strategies for merging data from many samples, which require assessment of sample isomorphism, rely on metrics of variability in unit-cell parameters, anomalous signal correlation and overall data similarity. Local scaling, anomalous signal optimization and data-set weighting, implemented in *phenix.scale_and_merge*, provide an efficient protocol for merging data from many samples. The protein NS1 was used in a series of trials with data collected from 28 samples for phasing by single-wavelength anomalous diffraction of the native S atoms. The local-scaling, anomalous-optimization protocol produced merged data sets with higher anomalous signal quality indicators than did standard global-scaling protocols. The local-scaled data were also more successful in substructure determination. Merged data quality was assessed for data sets where the multiplicity was reduced in either of two ways: by excluding data from individual crystals (to reduce errors owing to non-isomorphism) or by excluding the last-recorded segments of data from each crystal (to minimize the effects of radiation damage). The anomalous signal was equivalent at equivalent multiplicity for the two procedures, and structure-determination success correlated with anomalous signal metrics. The quality of the anomalous signal was strongly correlated with data multiplicity over a range of 12-fold to 150-fold multiplicity. For the NS1 data, the local-scaling and anomalous-optimization protocol handled sample non-isomorphism and radiation-induced decay equally well.

## Abstract 2

The benefits of using local scaling and optimization of anomalous signal (as implemented in *PHENIX*) for merging data sets from many crystals for determination of the substructure for weak anomalous scatterers are examined.

## 1. Introduction   

The use of high-multiplicity Bijvoet data from multiple samples has been shown to be effective in the determination of anomalous substructure in difficult cases (Akey, Brown, Dutta *et al.*, 2014 ▸; Liu *et al.*, 2012 ▸). These difficult cases include crystals with weak or few anomalous scatterers, poorly diffracting crystals and/or data collected far from the energy of peak absorption. While in principle increasing the multiplicity of merged data sets improves the data quality, complications arise from non-isomorphism when data from many samples are merged and from radiation damage when more data are collected from single crystals. Culling data, either by identifying and excluding outlier crystals or by removing the later diffraction images from radiation-damaged data sets, may be effective when merging data from many samples. Choosing an ‘optimal’ combination of data sets requires an assessment of individual data sets and, if radiation damage is suspected, a decision on how many data to include from each sample. Compatible data sets can be chosen by a variety of metrics including unit-cell variations (Foadi *et al.*, 2013 ▸; Liu *et al.*, 2012 ▸, 2013 ▸), anomalous difference and/or intensity correlation coefficients (Akey, Brown, Konwerski *et al.*, 2014 ▸; Giordano *et al.*, 2012 ▸; Liu *et al.*, 2012 ▸, 2013 ▸; Terwilliger, Hung *et al.*, 2016 ▸; Terwilliger, Bunkóczi *et al.*, 2016 ▸; Evans, 2006 ▸), and merging *R* factors (Evans, 2006 ▸).

A modification to data-merging strategies that relies on the weighting of data sets prior to merging has been implemented in *phenix.scale_and_merge* as part of the *PHENIX* suite of software (Terwilliger, Hung *et al.*, 2016 ▸). *Phenix.scale_and_merge* uses a multi-part strategy to optimize the accuracy of the anomalous differences in a merged data set. The strategy combines the use of an algorithm for local scaling, corrections for data anisotropy and, perhaps most importantly, a weight for each data set based on the correlation of anomalous differences to those of the average data. In principle, local scaling can ameliorate the effects of radiation damage, and weighting individual data sets allows the inclusion of more samples in the final merged data than does data culling. While *phenix.scale_and_merge* attempts to use as many samples as are available, outlier data sets are identified and excluded.

Here, we evaluate the strategy of local scaling and data-set weighting in comparison to the treatment of data with a global-scaling protocol. The system is the flavivirus NS1 protein, for which we solved the crystal structure from the anomalous scattering of sulfur (Akey, Brown, Dutta *et al.*, 2014 ▸). The S-SAD data are highly suitable for this experiment because complete data were recorded with a sixfold multiplicity of Bijvoet pairs for each of 24 crystals and with a threefold multiplicity for each of four other crystals. Previously, we reported that in certain instances better substructure solutions resulted when ‘outlier’ data sets were excluded (Akey, Brown, Konwerski *et al.*, 2014 ▸). This is confirmed when data are added (from best to worst) to create a merged data set (Terwilliger, Hung *et al.*, 2016 ▸). In the absence of accounting for inter-data-set variances, adding the ‘worst’ data sets degraded the accuracy of anomalous differences (Terwilliger, Hung *et al.*, 2016 ▸). The identification of outlier crystals can be tedious and may vary depending on the metric used. We tested whether the data-set weighting implemented in *phenix.scale_and_merge* would downplay the deleterious effects of slightly non-isomorphous crystals and eliminate the need to manually exclude data from outlier crystals.

We solved the crystal structure of the flavivirus NS1 protein by native sulfur SAD phasing at low resolution using multi-crystal data, followed by phase extension to 3.0 Å resolution, which yielded a high-quality electron-density map (Akey, Brown, Dutta *et al.*, 2014 ▸; Akey, Brown, Konwerski *et al.*, 2014 ▸). A total of 28 data sets were collected, 18 of which were combined for the original structure solution. Here, we investigate a local-scaling and anomalous-optimization strategy for weighting and combining individual data sets into a final high-multiplicity merged data set. As the focus is to optimize the determination of the anomalous substructure, the data analysis is limited to anomalous signal quality indicators in the low-resolution shells where an anomalous signal was detectable (4.0 Å). The metrics for assessing merged data quality were the correlation (CC_ano_) of observed anomalous differences (Δ*F*) with those from either an atomic model or a reference data set, the ratio of anomalous difference to total structure amplitude (〈|Δ*F*|/*F*〉) and the quality of the anomalous substructure determined from a merged data set using *SHELXD* (Sheldrick, 2010 ▸; number of sites found and CFOM). 〈|Δ*F*|/*F*〉 is a useful metric as it can be calculated in the absence of structural information, it appears to be a sensitive indicator of anomalous signal quality and it does not rely on error estimates. While the signal-to-noise estimate of anomalous differences [〈|Δ*F*|/σΔ(*F*)〉] could also be used, sigma values vary with the method used to estimate them and, especially for small differences and weak *F*, they may be an unreliable metric for evaluating different merging strategies.

## 2. Methods   

### 2.1. Data collection and processing   

As previously described (Akey, Brown, Konwerski *et al.*, 2014 ▸; Akey, Brown, Dutta *et al.*, 2014 ▸), data were collected from 28 individual crystals on beamline 23ID-D (GM/CA) at the Advanced Photon Source (APS), Argonne National Laboratory. As a compromise between minimizing sample X-ray absorption and maximizing sulfur anomalous signal strength, data were collected at 7.1 keV (Liu *et al.*, 2012 ▸), where the theoretical *f*′ is 0.365 e^−^ and the theoretical *f*′′ is 0.703 e^−^. At this energy, the estimated anomalous signal is approximately 1.5% (|Δ*F*|/*F*) based on the sulfur content (six disulfides and five methionines in 352 amino acids). In order to obtain complete anomalous data and to minimize systematic errors in anomalous differences owing to radiation damage, all data were recorded in an inverse-beam geometry (5° wedges) so that true Friedel pairs were recorded close in time. 2 × 90° of data (‘forward’ from goniometer setting 0 to 90° and ‘inverse’ from goniometer setting 180 to 270° in interleaved wedges of 5°) were collected for each crystal, with the exception of two samples (Nos. 152 and 155) for which only 45° × 2 were collected. Data were processed using *XDS* to a limit (*d*_min_) of 2.9 Å for all trials reported here (Kabsch, 2010*a* ▸,*b* ▸). For each crystal, the forward and inverse passes were processed separately but with common crystal-orientation parameters. A reference data set was assigned to ensure the indexing of all data with the same axis convention (space group *P*321). The forward and inverse passes for each crystal were scaled in *XDS* and output without merging.

#### 2.1.1. Assessing individual crystal data   

**Figure 1:**
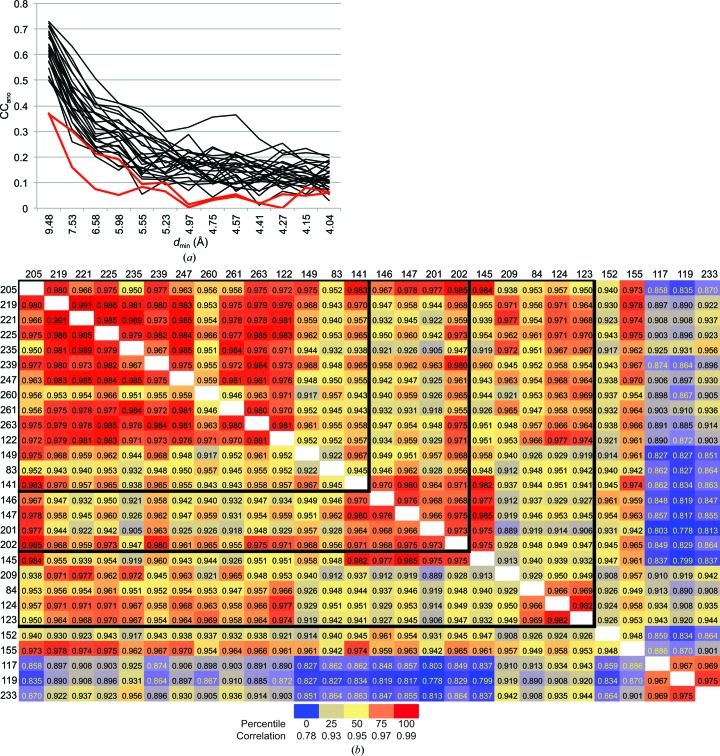
(*a*) CC_ano_ of individual data sets for 28 crystals compared with the reference 28-crystal merged data. *D*_ano_ from merged and scaled data (forward and inverse) for each sample was compared with *D*_ano_ from the complete, local-scaled 28-crystal data set. Two data sets (red traces; samples Nos. 152 and 155) are outliers. (*b*) Correlation coefficients (cumulative to a *d*_min_ of 3.5 Å) of structure-factor amplitudes (*F*) from scaled and merged data for each crystal (forward and inverse wedges combined) for each pairwise combination of samples. Data are coloured on a percentile scale (percentile ranking and correlation values are shown in the key) from blue (lowest correlation) to red (highest correlation). Bold lines separate the crystals of the 14-, 18-, 23- and 28-crystal data sets. This plot is symmetric about the diagonal.

For the current analysis, the 28 data sets from individual crystals were merged with *phenix.scale_and_merge* (using default settings and optimize_anomalous=True). Correlation coefficients (CC_ano_) of the observed anomalous differences for each crystal with the anomalous differences of a fully merged data set including all data from all crystals were calculated as a function of resolution (Fig. 1 ▸ *a*). The individual data sets were clustered with respect to CC_ano_, with the exception of the two samples for which only 45° of data were collected (Nos. 152 and 155). Pairwise correlations of amplitudes (*F*) were calculated for all possible crystal pairs (Fig. 1 ▸ *b*). This analysis identified three samples (Nos. 117, 119 and 233) that appeared to be outliers. Interestingly, these three crystals were centrally located in the narrow distribution of unit-cell parameters.

#### 2.1.2. Comparison of local to global scaling   

Data from 28, 23, 18 and 14 crystals were scaled and merged using both *AIMLESS* for global scaling (Evans & Murshudov, 2013 ▸) and *phenix.scale_and_merge* for local scaling (Terwilliger, Hung *et al.*, 2016 ▸). Data merged with *AIMLESS* used the scale keywords constant, bfactor on and brotation 20 to ensure true global scaling for these trials. The groups with fewer crystals were chosen as described previously (Akey, Brown, Konwerski *et al.*, 2014 ▸). Briefly, pairwise correlations among the 28 samples identified 18 that were deemed to be compatible and initially used to solve the NS1 structure. Retrospective analysis included testing all subsets of the 18 samples, of which four did not appear to contribute to substructure identification. Elimination of these four resulted in the 14-sample data set. Reprocessing of the initial ten outlier samples corrected mistakes in initial processing and brought five more samples into ‘compliance’. With the original 18, these compose the 23-sample data set. The five remaining outliers either had only 45° of data collected (Nos. 152 and 155; Fig. 1 ▸ *a*) or had poor correlation coefficients (*F*) to the remaining samples (Nos. 117, 119 and 233; Fig. 1 ▸ *b*). All samples had similar unit-cell parameters to within 1%, and those that deviated most from the average had a correlated deviation in the refined detector distance. Thus, unit-cell differences were not a useful determinant for data-set clusters in this case.

**Figure 2:**
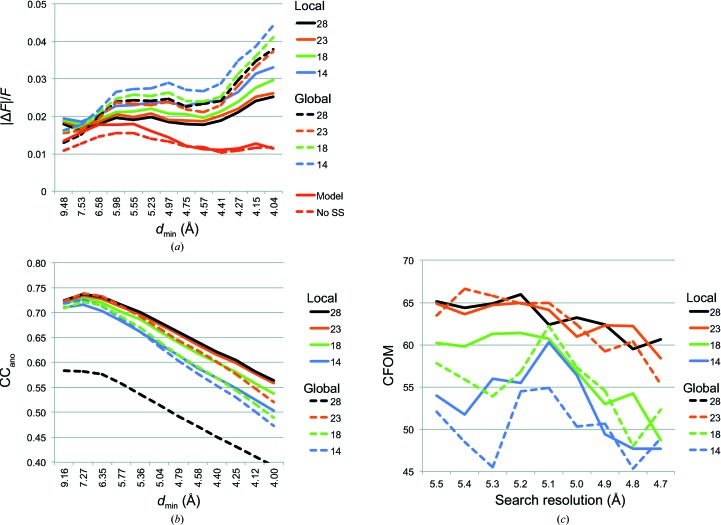
Comparison of local-scaled, anomalous-optimized data with global-scaled data. (*a*) Average fractional anomalous difference [〈|Δ*F*|/*F*〉] *versus* resolution for 28-crystal (black), 23-crystal (orange), 18-crystal (green) and 14-crystal (blue) data sets merged with *phenix.scale_and_merge* (solid lines) or *AIMLESS* (dashed lines). 〈|Δ*F*_calc_|/*F*_calc_〉 were from the refined model (PDB entry 4tpl) with intact disulfides (solid red lines) and with artificially broken disulfides (dashed red lines; S atoms were moved by changing each Cys rotamer). (*b*) Cumulative CC_ano_ between Δ*F*_obs_ for merged data and Δ*F*_calc_ from the refined model. (*c*) CFOM (CC_all_ + CC_weak_) of the best solution among 10 000 *SHELXD* trials at each limiting resolution. There is no plot for the global-scaled 28-crystal data because *SHELXD* failed to find any solutions.

Observed anomalous differences in the merged data sets were compared with calculated anomalous differences using 〈|Δ*F*|/*F*〉 (Dauter & Adamiak, 2001 ▸; Hendrickson *et al.*, 1985 ▸; Smith & Hendrickson, 2001 ▸) and CC_ano_, where CC_ano_ was calculated between the observed anomalous differences and the calculated anomalous differences based on the refined model (*phenix_refine* using PDB entry 4tpl and explicit sulfur *f*′ and *f*′′ values). For both metrics, the local-scaled, anomalous-optimized data were in better agreement with the calculated anomalous differences than were the global-scaled data (Figs. 2 ▸ *a* and 2 ▸ *b*). The average 〈|Δ*F*_calc_|/*F*_calc_〉 (Fig. 2 ▸ *a*, red curves) illustrates the increase in anomalous signal from disulfide scattering for data where individual S atoms are not resolved (solid red curve) compared with 〈|Δ*F*_calc_|/*F*_calc_〉 from a model where disulfides were artificially broken by rotation of the cysteine side-chain torsion (dashed red curve). In all cases, the 〈|Δ*F*_obs_|/*F*_obs_〉 were closer to expectation for local-scaled, anomalous-optimized data relative to the global-scaled data (solid *versus* dashed black, orange, green and blue curves), consistent with a lower level of noise in the anomalous signal for the local-scaled data. Although counterintuitive, a low value of 〈|Δ*F*_obs_|/*F*_obs_〉 for a data set from crystals with weak anomalous scattering indicates more useful anomalous differences than a high value because a high value reflects the errors in measurement of |Δ*F*_obs_| (Dauter & Adamiak, 2001 ▸; Hendrickson & Ogata, 1997 ▸). The CC_ano_ values were smaller in the lowest resolution shell than in the next lowest shell (Fig. 2 ▸ *b*). We surmise that this is owing to the difficulty in modelling bulk-solvent contributions to the overall structure factors, an effect that would have the largest impact at low resolution. Alternatively, systematic errors in measurement of the very low-resolution data may contribute. When using either local-scaled and anomalous-optimized or global-scaled protocols, addition of data from 14- to 18- and 23-crystal data sets yielded incremental improvements in the agreement with model anomalous differences (Fig. 2 ▸ *b*). However, inclusion of the ‘outlier’ data sets (from 23 to 28 data sets) had a marginal effect for the local-scaled data but severely degraded the anomalous signal with the global-scaled data. At higher resolutions, the local scaled data had a slightly better agreement with the model phases. Substructure identification (using *SHELXD*) was more robust for the 14- and 18-data-set combinations when using local-scaled and anomalous optimized data, with higher correlation coefficients (CFOM; Fig. 2 ▸ *c*). With 23 data sets, the local- and global-scaling protocols worked equally well, but when the ‘outlier’ data sets were included *SHELXD* failed to find any solution for the global-scaled data. We suspect that the data-set weighting in the anomalous-optimization protocol, rather than the local scaling *per se*, alleviated the poisoning of the combined data sets. 

#### 2.1.3. Merged data quality is strongly correlated with multiplicity   

**Figure 3:**
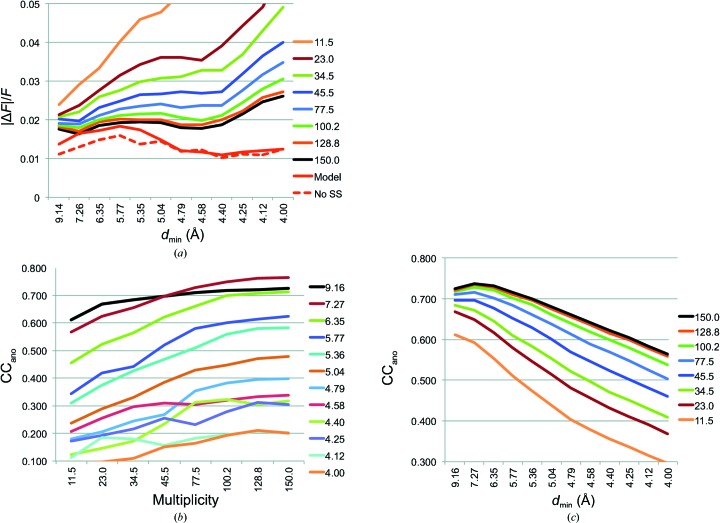
Metrics of anomalous signal for merged data sets from different numbers of crystals. (*a*) 〈|Δ*F*|/*F*〉 for local-scaled, anomalous-optimized data for merged data sets from two (11.5-fold multiplicity), four (23.0-fold), six (34.5-fold), eight (45.5-fold), 14 (77.5-fold), 18 (100.2-fold), 23 (128.8-fold) and 28 (150.0-fold) crystals with multiplicities indicated in the legend. (*b*) CC_ano_ (Δ*F*_obs_ compared with Δ*F*_calc_) by resolution shell as function of increasing multiplicity, shown for individual resolution bins. (*c*) Cumulative CC_ano_ for data sets of variable multiplicity.

The merged data sets from 28, 23, 18 and 14 crystals have an unusually high multiplicity of anomalous differences (78-fold to 150-fold). We expanded the analysis to merged data sets with more typical multiplicities ranging from 12-fold to 45-fold. Merged data sets were generated from eight, six, four and two crystals that were chosen based on the highest correlation of Δ*F* with the 28-crystal reference data set (Fig. 1 ▸). As expected, the anomalous signal quality steadily decreased with decreasing multiplicity (Fig. 3 ▸). Additionally, data combinations using fewer than 14 crystals failed in anomalous substructure determination with *SHELXD* (Table 1 ▸).

#### 2.1.4. Local-scale handling of radiation damage and non-isomorphism   

**Figure 4:**
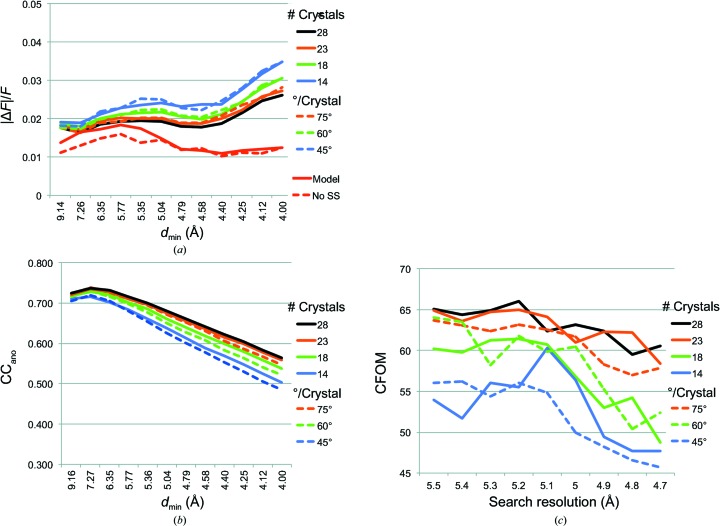
Comparison of damage-minimized and crystal-reduced multi-crystal data sets. (*a*) 〈|Δ*F*|/*F*〉 *versus* resolution for 28-, 23-, 18- and 14-crystal data sets (solid lines) and damage-minimized 75, 60 or 45° data sets (dashed lines) of matched multiplicity. In red are calculated values for 〈|Δ*F*|/*F*〉 from the refined model. (*b*) Cumulative CC_ano_ comparing Δ*F* for merged data with those calculated from the refined model. (*c*) CFOM (CC_all_ + CC_weak_) of the best solution from 10 000 *SHELXD* trials at each limiting resolution.

Both non-isomorphism and radiation damage have deleterious effects on the ability to estimate accurate anomalous differences for multi-crystal data sets. These effects are expected to differ in magnitude and to be dependent on the details of data collection and on the crystals. Thus, the effect on signal quality when multiplicity is reduced may depend on whether the reductions were achieved by limiting the number of crystals or by limiting the number of experimental observations per crystal. In the case of NS1, a total of 180° of data were recorded from nearly all crystals (90° for each of the forward and inverse passes, recorded in interleaved 5° wedges). The diffraction images from the end of the data collection for each of the 28 crystals exhibited a visible reduction in the diffraction limit, which was up to 2.9 Å at the start of data collection and typically decreased by 0.5–0.8 Å by the end of data collection. Thus, we anticipated that excluding the data most affected by radiation damage, *i.e.* the forward and inverse images recorded last in the data collection, might improve the anomalous signal relative to data sets where an equivalent multiplicity was achieved by excluding crystals. We created ‘damage-minimized’ data sets for all 28 crystals by excluding the last-recorded 15, 30 or 45° of data and then merged these data with local scaling. Each of these ‘damage-minimized’ merged data sets was compared with a data set of similar multiplicity that included the full 90° of inverse-beam data from fewer crystals [the first 75° of data from 28 crystals (127.5-fold multiplicity) *versus* 90° of data from 23 crystals (128.8-fold multiplicity), the first 60° of data from 28 crystals (104.6-fold multiplicity) *versus* 90° of data from 18 crystals (100.2-fold multiplicity), and the first 45° of data from 28 crystals (80.2-fold multiplicity) *versus* 90° of data from 14 crystals (77.5-fold multiplicity)]. The full 90° data set from 28 crystals had a 150.0-fold multiplicity of anomalous pairs. At equivalent multiplicity, the anomalous signal metrics were roughly equivalent for the damage-minimized and the crystal-reduced data sets (Fig. 4 ▸, Table 1 ▸). This suggests that to a first approximation and in the case of the NS1 data, discrepancies owing to non-isomorphism and discrepancies owing to radiation damage were handled equally well by the local-scaling protocol. Nevertheless, we note that the |Δ*F*|/*F* and CC_ano_ metrics are slightly but consistently better for the reduced-crystal data sets than for the damaged-minimized data sets with matched multiplicity.

## 3. Summary   

In principle, local scaling will better account for intensity differences that arise either from radiation damage or from errors in modelling sample absorption. Here, data for each of several crystals were locally scaled and individual crystal data sets were merged with anomalous optimization. This scaling-and-merging procedure resulted in a merged multi-crystal data set with lower noise in the anomalous differences than was observed for the same multi-crystal data that were scaled globally and merged without anomalous optimization. Local scaling resulted in more accurate estimates of anomalous differences at lower data multiplicity while data-set weighting to optimize anomalous differences down-weighted suspect data (23- *versus* 28-crystal data sets), thus minimizing the need to cull data from a combined meta-data set. Both local scaling and data-set weighting contributed to greater success in substructure determination.

The most striking result of our investigation is that the overall quality of anomalous differences was similar for data sets of equal multiplicity, whether data multiplicity was reduced by excluding crystals or by excluding the most radiation-damaged segments of data from each crystal. For each of three multiplicities tested (128-fold, 100-fold and 80-fold), the crystal-reduced data were slightly superior to the damage-minimized data acccording to the criteria 〈|Δ*F*|/*F*〉 and correlation to calculated anomalous differences. We had anticipated the opposite: that excluding data to minimize the effects of radiation damage would result in a substantial improvement in the quality of anomalous differences. Two effects may contribute to the finding that achieving a given multiplicity by using fewer crystals was slightly better than by using less data from each crystal. NS1 crystals diffracted to ∼3 Å resolution, but we analysed only the strongest data (to 4.0 Å resolution) as the anomalous differences beyond this limit were not useful. The lowest resolution data are expected to be least influenced by radiation damage, so that the anomalous differences to 4.0 Å resolution may be preserved despite visible damage at the ∼3 Å limit during the 90° (×2) of data collection from each crystal. The second effect is a reduction in the multiplicity of observed anomalous differences within each crystal for the damage-minimized data sets (from sixfold in the full 90° data sets to threefold in the 45° data sets). For weak anomalous differences at low resolution (estimated to be 1.5% of *F* for NS1; solid red curve in Fig. 4 ▸ *a*), the greater multiplicity was evidently superior to the avoidance of the apparently modest effects of radiation damage at 4.0 Å resolution and below. This is also clear in the pairwise CC_ano_ values, where anomalous differences in the data of the two crystals from which only 45° (×2) of data were recorded were far more poorly correlated to anomalous differences from other crystals even though the amplitudes (*F*) were well correlated.

Anomalous substructure-determination relies almost exclusively on low-resolution, typically high-intensity data, and we have seen that local scaling and anomalous optimization improve the quality of anomalous differences in multi-crystal data sets. This may not be the best approach to generate structure-factor amplitudes to the diffraction limit of crystals, as local scale factors in high-resolution shells are generated using only the weakest data. A more global scheme may be more suited to preparing a multi-crystal data set for model refinement. The combination of local scaling and anomalous optimization appears to provide a robust and efficient method for combining data from many crystals to maximize the quality of the anomalous signal in difficult problems.

## Figures and Tables

**Table 1:** Correct sulfur sites found (of 23 possible sites: 12 S–S, ten Met and one SO_4_^2−^) with *SHELXD*

Multiplicity (anomalous)	Scaling protocol	No. of crystals	Data per crystal (°)	Sites found
150	Local	28	90	20
150	Global	28	90	Failed
129	Local	23	90	20
129	Global	23	90	20
128	Local	28	75	21
105	Local	28	60	19
100	Local	18	90	20
100	Global	18	90	20
80	Local	28	45	19
78	Local	14	90	19
78	Global	14	90	16
45	Local	8	90	Failed
